# “Unless someone sees and hears you, how do you know you exist?” Meanings of confidential conversations – a hermeneutic study of the experiences of patients with palliative care needs

**DOI:** 10.1186/s12912-024-01988-9

**Published:** 2024-05-18

**Authors:** Tove Stenman, Ylva Rönngren, Ulla Näppä, Christina Melin-Johansson

**Affiliations:** Department of Health Sciences Nursing Science, Mid Sweden University, Östersund, S-831 25 Sweden

**Keywords:** Confidential conversations, Communication, Hospice, Home care, Nursing, Palliative care, Qualitative research

## Abstract

**Background:**

Patients with palliative care needs live with the reality of limited time due to illness or age, eliciting emotional and existential responses. A failure to address their existential needs can lead to significant suffering. A person-centred approach is paramount to effectively address these needs, emphasising holistic care and effective communication. Although existing communication models focus on predefined frameworks, a need exists to explore more spontaneous and confidential conversations between patients and nurses. Confidential conversations have the potential to build therapeutic relationships and provide vital emotional support, highlighting the need for further research and integration into palliative care practice. This study aims to more deeply understand the meaning of confidential conversations for patients with palliative care needs.

**Methods:**

In-depth interviews were conducted with 10 patients in the context of specialised palliative care. A hermeneutic analysis was used to gain a deeper understanding of the meanings of the conversations.

**Results:**

The patients had varying experiences and wishes concerning confidential conversations. They strived for self-determination in finding confidants, seeking trust and comfort in their interactions with nurses. Trust was crucial for creating a safe space where patients could express themselves authentically. In shared belonging, confidential conversations with a nurse provided validation and relief from life’s challenges. Experiences of feeling unheard or rejected by a nurse could intensify loneliness, prompting individuals to withdraw and remain silent. Regardless of the motives behind their choices, it was crucial that patients felt respect and validation in their decisions. Their autonomy could thus be recognised, and they felt empowered to make decisions based on their unique preferences.

**Conclusions:**

Patients value trust and understanding, particularly in confidential conversations with nurses, which offer solace, validation and empowerment. However, indifference can increase patients’ suffering, fostering self-doubt and reluctance to engage further. To address this, health care can prioritise empathic communication skills, offer ongoing support to nurses, and promote continuity in care through investment in training and resources. Additionally, adopting a person-centred approach in confidential conversations is crucial, considering patients’ varying preferences.

**Supplementary Information:**

The online version contains supplementary material available at 10.1186/s12912-024-01988-9.

## Background

Patients with palliative care needs live with the awareness that time is limited due to illness or advanced age [1]. Living with the uncertainty of life’s duration may evoke a range of reactions, emotions, and existential thoughts [2]. Death is difficult to come to terms with, and if a person cannot accept the reality of mortality, a risk of existential suffering arises [3]. Patients employ diverse strategies to handle their forthcoming death [2], one of which involves engaging in conversations with family, friends, and healthcare providers [1, 2]. Conversations based on the individual patient’s specific needs, wishes, abilities, and conditions may be healing and even therapeutic [4]. Research indicates that engaging in conversations to express thoughts and emotions could benefit older people’s well-being [5]. Patients with palliative care needs strive to live a meaningful life. Focusing on living requires support from the healthcare system, including healthcare professionals, the environment, and the organisation of palliative care [6].

A person-centred approach is essential for addressing patients’ individual needs and goals. Essentially, person-centeredness embodies an ethical stance guiding both patients and professionals toward fostering healthful relationships within health care [7]. Palliative care philosophy is by nature person-centred and emphasises a holistic approach to the person and the goals of the hospice philosophy. This holistic approach addresses physical, psychological, social, and existential suffering and well-being [1]. A central approach involves communication and interaction between patients and nurses, grounded in patients’ beliefs, needs, stories, thoughts and feelings [8, 9]. A person-centred approach may ensure holistic care by nurses to patients with palliative care needs [10]. Conversation between patients and nurses is anything but superficial; it is complex, effective, and confidential, offering important potential for interaction and emotional support [11]. Spontaneous and natural conversations are rarely described as they occur alongside other care activities, their apparent simplicity rendering them easily overlooked and invisible [12, 13], however, they have potential and a unique opportunity to take advantage of support for patients with palliative care needs [12]. Patients with palliative care needs report receiving insufficient emotional and existential support [14–17]. This lack of support is associated with higher psychological stress and lower well-being [14, 17, 18].

In conversations, the nurse can support the individual patient’s specific needs of alleviation, and conversations are thus key within the fundamental framework of palliative care [1]. Conversations in palliative care that involve healthcare professionals are described as palliative care communication [19], advanced care planning [20], serious illness conversations [21] and existential conversations [22]. These different models of communication are not separate but can flow into each other, effectively meeting patients’ and relatives’ needs and preferences. Open and honest information, empathy and dedicated healthcare professionals contribute to effective communication. Open and honest communication may also arouse patients’ anxiety, stress and existential concerns [23].

Common to the conversations described is that either they are initiated by healthcare professionals and aim to exchange or obtain information or they have a predetermined purpose or content. Some communication models primarily focus on physicians and do not include the valuable communication skills that nurses possess. Therefore, further research is needed on spontaneous, naturally occurring communication by nurses in palliative care [23]. It is indicated that in a confidential conversation with a nurse, the atmosphere can be perceived as natural and spontaneous. Here, the patient can confide in the nurse about their wishes and thoughts. These interactions could facilitate the development of therapeutic relationships, allowing patients to express their thoughts, regardless of what they could be and gain support in their situation [24].

Confidential conversations and their content in the context of palliative care are under development as a research area and are further explored and developed in this study, grounded in patients’ perspectives and experiences. Complementary to other communication approaches, confidential conversations may be relevant to address patients’ emotional and existential concerns where support may be lacking. To enhance and expand communication as a fundamental aspect of palliative care, focusing on the significance of confidential conversations is crucial. Furthermore, this study intends to deepen the knowledge of the approaches that patients use in confidential conversations with nurses in the context of palliative care.

## Methods

### Aim

To more deeply understand the meanings of confidential conversations for patients with palliative care needs.

### Design

To develop a deeper understanding, a qualitative hermeneutic design inspired by Geanello’s [25] method was used. The analysis brings into the study Ricoeur’s theory of distanciation as part of the hermeneutic philosophy of understanding. Ricoeur’s hermeneutic theory aligns with the philosophical foundations of interpretive research and focuses on the relationship between ontology (the interpreter) and epistemology (interpretation). It allows researchers to use hermeneutic approaches to textual analysis, which is essential for understanding complex human experiences in the context of nursing [25].

### Setting and participants

The setting for the study was palliative care units in a sparsely populated region in Northern Sweden, comprising four counties with a total of approximately 900,000 inhabitants [26]. The catchment area included two hospices, one hospital ward with round-the-clock care, and nine home health teams providing specialist palliative care [27].

Four specialist palliative care units providing care at home or in hospice participated One facility was excluded due to long travel distances and short treatment times, i.e. the enrolled patients were seriously affected by their illnesses. The patients received verbal information about the study from a unit manager, and the research group was notified of the patients´ contact details after their consent. Patients over 18 years old, who understood the meaning of participation and could participate in an interview of around 30 min could be included. A total of 19 patients were purposively sampled, and nine were excluded due to rapid deterioration, of whom three died. A total of 10 patients participated, six women and four men, aged 56–85 years (m 70) with advanced cancer. Nine were cared for in their homes by specialist palliative care teams, and one was cared for in a hospice. The performance status of the patients was 2.5 on the Eastern Cooperative Oncology Group Scale of Performance Status (ECOG PS). This indicates that the patients were confined to a bed or chair for more than 50% of their waking hours. The ECOG PS describes a patient’s level of functioning in daily life from 0 to 5, where 0 is fully functional and 5 is death [28].

### Data collection

To enhance the research comprehension and generate novel insights, the research group conscientiously reflected on their positionalities and reflexivity both before and during the data collection and analysis. All researchers were registered nurses with extensive professional experience in fields where interpersonal conversations hold significant importance, including palliative care and psychiatric care. Recognising and acknowledging one’s pre-existing understandings is crucial, because it ensures that the subject is approached with appropriate depth and openness to novel perspectives. Awareness of one’s pre-understanding is important, giving the subject its proper dimensions and new perspectives [29].

Data were collected from November 2022 to February 2023 using qualitative interviews and demographic information questions (Supplementary File 1). The interviews were conducted by TS and YR with the support of an in-depth interview guide that contained open and follow-up questions to encourage the participants to reflect and deepen their thoughts [30]. Nine patients were interviewed face-to-face, and one was interviewed by telephone. The interviews began with a brief introduction of the interviewer and verbal information about the study, including that participation was voluntary. The interview prompts included: ‘Please, tell me about an occasion when you felt you had a confidential conversation with a nurse’. Follow-up questions and prompts were used, such as: ‘Can you elaborate?’ If they had not had a confidential conversation with a nurse, the follow-up question was whether they could speak more about that.

The interviews lasted between 22 and 77 min (m 36 min) and were digitally recorded. The visits at the patients’ residences lasted between one and three hours. To ensure reliability and dependability, the interviews were transcribed verbatim. The data were stored on a password-protected server at Mid Sweden University.

### Data interpretation

The analysis was based on Ricouer’s method with distinction: explanation (content) and understanding (meaning). In the transition from speech to written text, the dialogue is made into writing, where meaning becomes more important than the words and allows for deeper interpretation. The text becomes autonomous and open to interpretation, overcoming the limitations of face-to-face dialogue [25].

In the first step, the interviews were transcribed and the text was organised. Repeated naïve reading initiated a preliminary interpretation, and new questions were raised and asked about the text. From part to whole, and from whole to part, an understanding crystallised. The hermeneutic circle characterizes this process. Returning to the data, the whole and the pre-understanding merged. Finally, a critical discussion of the themes was based on the pre-understanding of the research group. The analysis gave us a new understanding through the explanations of the text, and our horizons merged and expanded. Table 1 shows examples of the analysis process.

**Table 1:** (Examples from the analysis process)

Meaning units	Subtheme	Theme
If I were to have those thoughts in such a situation, then I wouldn’t expect that these are questions that the nurse is a specialist in, but in that case, I would probably prefer that I … if I … talk a little about what I think and feel, so I think the natural answer would be that then we would see if we can connect you with another person in this, who can provide the answers because you also want the nurse to start answering questions that she really isn’t sure. So I think it’s a … it’s a real attitude if they do that if they lead one to someone else so to speak…	To select an appropriate dialogue partner	Being self-determinant in finding confidants
… I need to feel… safety … that I am safe and that they hear and listen to me, that they hear what I want and what I need. And… and… and that they can say exactly… so also to me, that you can say exactly as it is, without feeling: “Do I dare to say this?” …however, I feel completely safe with the nurses that I have.	To seek trust in interpersonal relations	Being self-determinant in finding confidants
I don’t know if I… I do tend to chat quite a bit, but I rarely delve too deeply. Those… I’ll probably keep to myself. There are things I just don’t discuss… But I won’t know until I reach that point. Yes, I’ve had someone build a sort of wall around me, even though I’m quite open. It sounds odd. No…no… It’s… it’s… I’m not sure how… I suppose I’ve lived like that all my life… that I… Yes. The most personal things, I keep to myself. And it takes a lot for me to open up. I think I would have just brushed it off if it had been an attempt at conversation… It’s probably me not wanting to. This is how I want my… life to be.	To preserve integrity	Being self-determinant in finding confidants
And she kind of came up with the last piece of the puzzle which was… which she put down… it so gently. “You are so sick. This is so sad”… in a way…she said it in a way…and it was so nicely said, so I was just: yes, I am. And then I kind of got this chance to… yes… and then ask her can you sleep now, she said? Yes, I can, because I feel so calm … very calm I felt, because she was so … so calm and we talk about … and she came in with medicine … I was in a lot of pain and then the pain went away and then she says that and it turned out like this… yes… yes … it was… yes, but it was a very nice experience…	To find confirmation	Being in shared belonging
It’s the whole package that is… And then she was also a bit joking, now you want… you don’t want to have only this serious all the time, but something small… some joke sometimes like that, that’s the way it should be, I think. It is, it is hard as it is, and then it can be fun with a little joke or how to insert something like this which is… yes. No, if you’re going to say how you felt, then it felt like she wished me well.	Finding respite in the moment	Being in shared belonging
…and staff who come in and are bitter and in a bad mood…that makes the whole atmosphere in my room… then…there is only negative air and energy in the room…and that makes me just…sad… that… and it’s great… I can’t demand that everyone thinks that life is fun, to come to me or that work is fun, but sometimes you make the hell out of me, excuse the expression… Now now … I suppose you can feel it… but now you find nurses and such, but… but… but… It almost hurts the heart… if you need to talk to someone and then you are… Rejected.	To feel rejected and lonely	Being in-between
… So, when they’re under such stress, you notice it immediately because they’re rushing back and forth, and there are alarms and beeps and all that… and then I feel like you don’t want to interrupt by trying to talk about something. But you just… you’ve already decided not to speak, because you see that: There won’t be… there’s no time or opportunity to speak here, so I stay silent instead.	To become silent if no one is listening	Being in-between

### Ethical considerations

Ethical approval for the study was obtained from the Ethical Review Authority in Sweden (Dnr 2021–04066 and 2022-03769-02). Ethical considerations followed the research rules presented in the Declaration of Helsinki [31].

Even though patients with palliative care needs are in a vulnerable situation, they must be invited to participate in research. Ethical considerations are particularly important when collecting data because prognosis, energy and ability are considered, with flexibility for individual needs. A sensitive approach characterised by empathy was necessary for inclusion, but it also considered the patient’s self-determination [32, 33].

Written consent was collected from operations managers, and written and oral consent was collected from the patients. They were informed that their participation was voluntary and that they could drop out of the study without explanation. The researchers performing the interviews had no professional relationship with the participants. The participants’ identities were coded, and transcribed material was stored in a locked cabinet. The unit manager would be contacted if the patients wanted support due to strong emotions during the interview.

## Results

The patients had varying experiences and requirements concerning confidential conversations. Some expressed a strong desire to talk and verbalise their thoughts, whereas others preferred to refrain from sharing their innermost thoughts with nurses. For some, remaining silent was their method of handling intense emotions, whereas others did not feel confident enough to disclose themselves in confidential conversations with a nurse. Regardless of the motives behind their choices, it was crucial that patients felt respect and validation in their decisions. Their autonomy could thus be recognised, and they felt empowered to make decisions based on their unique preferences.

In the analysis, three themes and seven subthemes emerged, All themes were interconnected and are presented in Table 2.

**Table 2:** Overview of themes and subthemes related to the meanings of confidential conversations for patients with palliative care needs

Theme	Subtheme
Being self-determinant in finding confidants	- To select an appropriate dialogue partner
	- To seek trust in interpersonal relations
	- To preserve integrity
Being in shared belonging	- To find confirmation
	- To find respite in the moment
Being in-between	- To feel rejected and lonely
	- To become silent if no one is listening

### Being self-determinant in finding appropriate confidants

In the confidential conversations, patients felt vulnerability and apprehension when expressing their deepest thoughts and emotions. Such revelations required a foundation of trust, leading patients to carefully select whom they confided in. They adeptly navigated various support structures and relationships to find the right confidant, whether seeking professional assistance or turning to trusted relatives or friends. This decision reflected a profound awareness of their need for trust and understanding.

#### To choose an appropriate dialogue partner

The patients highlighted the pivotal role of interpersonal skills by expressing an awareness of nurses’ communication abilities. They showed deep sensitivity to the nuances of verbal communication competence and demonstrated a genuine empathy for the challenges that nurses might face in engaging in confidential conversations. This had implications for the choice of confidant. One patient expressed this sentiment, saying: “…some nurses couldn’t talk… I noticed it right away… and I felt like this; I knew there were other nurses, so it didn’t matter.” (patient no. 207).

The patients perceived that some nurses were not feasible as dialogue partners due to their heavy workload. Their understanding extended beyond mere recognition of workload; they empathised with the nurses’ demanding work and the complexities of their roles. They accepted and respected the limitations that nurses faced. These encompassed factors beyond a heavy workload to include resources and time constraints, which could lead to emotional and psychological demands. The patients understood that these constraints did not indicate a lack of willingness to provide support. One patient said: “I find myself thinking, ‘Should I do this? Should I… maybe I shouldn’t… ask about this,’ especially when I sense the stress around me… I can save it for another time.” (patient no. 204).

The patients also did not expect the nurses to fulfil all their conversational needs. They recognised the broader context in which nurses operate. They acknowledged that nurses had multiple responsibilities, and they had a deep understanding of them being a part of a larger healthcare team with diverse responsibilities. This understanding reflected empathy towards the challenges that nurses faced and a realistic perspective of the healthcare system’s complexity.

In addition to formal care settings, patients sought support from personal networks outside the healthcare system. They leaned on family members, close friends and other professionals for alternative forms of support and confidential conversations. Within these networks, patients found the support they sought, underscoring their deliberate choice to seek assistance from sources they apprehended as capable of fostering meaningful and supportive interactions. This conscious decision underscored their commitment to prioritising relationships that could facilitate understanding and empathy.

#### To seek trust in interpersonal relations

The patients expressed that they sought special trust and trustworthiness in their confidant and that it required some form of relationship. They exhibited autonomy when considering issues that resonated with their unique experiences and needs. Trust played a central role, fostering a sense of security and comfort in sharing their innermost thoughts and emotions. One patient argued that:

Yes, maybe it could be that the more often you meet the same person, that that’s what’s important, that you get to meet the same person… yes, I could talk to them… then we talked about children and all sorts of things … But just that, I think that it’s important that you get to have … not have so many different people around you, then you get that feeling of trust. (patient no. 206)

Establishing trust within interpersonal relationships with nurses was pivotal for selecting a confidant, whether gradually developed or established in a single encounter. Furthermore, the initial interaction with a nurse significantly shaped the patient’s perception of trust, indicating that trust could be established in the immediacy of the encounter. Deepening trust within interpersonal relationships not only facilitated the selection of a confidant but also created an empowering atmosphere where patients felt comfortable having confidential conversations. This emphasised the profound impact of trust in interactions, as one patient confirmed:

I trust everyone who comes, but it’s difficult for me to open and talk freely… However, it is possible to talk… if I want to… I can talk to everyone who gets here. It’s reassuring when they’re [the nurses] attentive, but I don’t always feel the need to talk. However, I do feel comfortable talking with anyone in the staff. (patient no. 203)

Patients desired nurses to be attentive and available, ready to engage in confidential conversations when the patients felt comfortable. Creating a supportive environment and a solid relationship where patients felt empowered to express themselves authentically without fear of judgment or coercion was essential.

#### To preserve integrity

The intricate relationship between patients’ integrity and their willingness to share intimate thoughts and emotions was highlighted. Some reluctance was expressed to talk about personal feelings; exposing such vulnerable aspects of themselves felt unnecessary and even contrary to their sense of self. This sentiment was captured by one patient who had never before opened up to an outsider about deeply personal matters: “I think… I’ve never talked… with an outsider about personal things, that is, deeply personal things. I’ve never talked to anyone about it… Never. I don’t need it…” (patient no. 208).

Patients found vocalising personal issues overwhelming or unnecessary at times. Their reluctance to engage in conversation did not necessarily signify avoiding confrontation of their situation or rejecting support from nurses. Instead, it served as a protective barrier, allowing patients to maintain a sense of control and integrity amid vulnerability. It could also mean that they desired to be in solitude with their thoughts, which served as a means of preserving personal integrity. It provided a space for introspection and emotional processing.

Respecting the patients’ decisions regarding the initiation of confidential conversations was crucial. Choosing not to engage did not indicate a lack of need for support or understanding; rather, it reflected their preferences for navigating and processing their experiences while preserving their integrity.

### Being in shared belonging

Patients found solace and validation in confidential conversations with nurses. The patients felt seen, understood and empowered by the nurses, which led them to express themselves authentically. These interactions offered relief from the challenges of illness and fostered emotional support, highlighting the importance of trust and genuine connection in relationships.

#### To find confirmation

The patients experienced a profound sense of confirmation in the confidential conversation and relationship with the nurse. The patients’ existence and experiences were not only acknowledged but also validated. In the confidential conversations, patients felt truly seen and understood as they articulated their innermost thoughts and emotions. This validation enhanced their sense of worth, reinforcing the belief that their voices mattered, as one patient stated: “So, it felt like… it felt like she [the nurse] thought it was important in some way, what I had to say” (patient no. 206).

Central to the success of these conversations was how nurses responded, and this success played a critical role in nurturing and maintaining patient–nurse relationships. The feeling of being confirmed and validated in the confidential conversation was strengthened by being met with genuine curiosity and openness from the nurse. One patient expressed it as:

They might have been running around, busy as could be, but still found a few minutes to spare for a chat… maybe just 2–3 min, but those brief moments meant the world to me. It was like, ‘Oh, how comforting, they made time for me.’ It was a tangible reminder of their presence. (patient no. 202)

This created an environment where patients felt comfortable and encouraged to authentically share their thoughts and feelings. By feeling validated and sensing genuine interest in their well-being, a sense of trust and connection was established, allowing meaningful and honest dialogue to develop naturally. Crucially, patients felt confirmation across the entire spectrum of emotions and subjects, unrestricted by judgment or devaluation. They found solace and affirmation in discussing even the most challenging topics, knowing that no subject was deemed too daunting or uncomfortable. As one patient expressed, “…when I share my innermost thoughts, however silly or ridiculous they may seem at times… to express them to someone, it holds significant meaning, allowing me to unburden myself…” (patient no. 208). This highlighted the transformative power of affirmation within the context of confidential conversations.

#### To find respite in the moment

Engaging in trusting conversations with a nurse went beyond simple dialogue; it provided patients with a break from the challenges of illness and forthcoming death. Within these conversations, patients found a space where they were seen as complete individuals with their hopes, fears and dreams, rather than just being defined by their medical conditions. This respite was not transient; it was a sanctuary where they felt understood and accepted, shielding them from the existential uncertainties such as fears related to death, loss of control and sorrow that often accompany serious illness. The patients felt empowered to share their innermost thoughts and feelings without fear of being judged or criticised, in the presence of trusted nurses. This allowed them to express themselves authentically, fostering a sense of empowerment. This was explained by one patient:

A nurse who stayed with me during a difficult conversation, even as I stood there, tears streaming down my face [cries] while discussing my illness. She simply stood there and stood and stood… listening attentively, unwavering. It was an incredible moment for me, a sense of relief washing over me, as if to say, ‘Now I can handle this [breathes out], I can manage.’ (patient no. 205).

Additionally, moments of shared laughter and joy provided patients with brief yet significant relief from their vulnerable situation. These confidential conversations became profound acts of validation and existential affirmation, offering a break and emotional support. The impact of such conversations is aptly captured in the following quote:

Yes, having someone who can listen and share a bit of the burden with you is truly comforting. It’s reminiscent of the care you received as a child when a mother figure would step in during tough times. It’s incredibly reassuring. (patient no. 209)

This sentiment underscores the profound comfort and reassurance that patients derive from these trusting interactions.

### Being in-between

Being rejected when feelings and thoughts were expressed and the patient was vulnerable increased the feeling of loneliness. Opting not to start a confidential conversation following such experiences was not merely about avoiding discomfort but also a way to protect themselves. This meant trying to handle the complex feeling of wanting to have a confidential conversation but at the same time not being listened to or understood, a feeling of being in-between. This inner conflict discouraged patients from revealing themselves in confidential conversations with a nurse.

#### To feel rejected and lonely

Patients recounted situations where their attempts to communicate with nurses were met with indifference or dismissive remarks, which led to feelings of rejection and loneliness. These encounters left them with a sense of isolation and alienation as they struggled to find validation and understanding in their time of need. The feeling of being rejected was grounded in not feeling seen or listened to when they needed to talk, explained by one patient: “…I can’t expect every nurse to share my perspective on life or work, to come to me with enthusiasm. It’s just something you sense… It’s almost heart-breaking… if you need to talk to someone and then you’re… rejected.” (patient no. 202).

The patients felt neglected, which could lead to increased suffering when the nurse did not continue the conversation or they were met with uninterest. The absence of meaningful dialogue in these moments had significant existential implications, prompting patients to question their existence and purpose. They felt unheard and their needs unmet without acknowledgement and validation from the nurses.

These experiences of rejection and neglect could induce feelings of shame and guilt for burdening others with their difficulties. Despite their longing for connection and support, patients felt compelled to step back because they considered themselves burdensome rather than deserving of care and attention. One patient put it like this:

Yes… but I’ve encountered… well, they are nurses too, some who can be a bit snarky. Or who perhaps speaks a little too loudly. So, you notice these things, you notice them… and then I wonder… Is it because what I’m sharing is too heavy or not? (patient no. 204)

These encounters with indifference and dismissal not only left patients feeling rejected and lonely but also challenged their sense of self-worth. Beyond the absence of dialogue, it was a feeling of being unseen and unheard precisely when they needed acknowledgement the most. One patient said: “Unless someone sees you and hears you, how do you know you exist and why should you exist at all?” (patient no. 205).

#### To become silent if no one is listening

The experience of feeling rejected or neglected could deter the patient from attempting to initiate conversation again. Remaining silent after feeling silenced extended beyond a mere momentary response; it signalled a reluctance to seek help or a loss of confidence in the nurses’ ability to comprehend their needs. This loss of confidence also led patients to question the validity of their experiences and needs, fostering feelings of self-doubt and insecurity:

…Because it’s painful, you know, when you find yourself in situations like this, it’s like you start questioning yourself, feeling a bit unsure. How much can I ask for? Do I dare to speak up, or am I bothering? …But even then, you still can’t help to feel a bit hesitant, thinking, ‘Should I say this?’ (patient no. 204).

Maintaining silence was expressed as a form of self-preservation in vulnerable circumstances. Patients struggled with internal conflict, yearning to express themselves while simultaneously feeling unheard and unacknowledged. This conflict stemmed from a desire to address difficulties while safeguarding their vulnerability. This realisation highlighted a subtle yet profound disconnection as patients perceived the nurse’s lack of acknowledgement or recognition. Consequently, they hesitated to engage further in conversations, fearing continued neglect despite their escalating needs and suffering. This hesitation eroded the trust placed in the nurse, potentially deepening the detachment in the patient–nurse relationship. Such feelings could dissuade patients from seeking further dialogue. The following quote encapsulates the feeling of being dismissed:

Because… if I show… if I put my hand out and say, ‘I think it’s difficult today’… I feel so damn bad, and then [the nurse] says, ‘Well, what a shame,’ and then they leave… then I shut up. (patient no. 202)

Feeling rejected or neglected could discourage patients from initiating further conversation and cause them to remain silent. This reluctance reflected a loss of confidence in the nurse’s ability to understand their needs and led to feelings of self-doubt and insecurity.

## Discussion

This study aimed to more deeply understand the meanings of confidential conversation for patients with palliative care needs, an area of limited research. The findings underscore the importance of patient autonomy in selecting dialogue partners and shaping conversation dynamics. The patients strategically navigated support networks to find suitable confidants within and beyond healthcare settings.

It is indicated that confidential conversations, from a nursing perspective, occur at the patient’s initiative, often spontaneously, and with unforeseen content [24] and thus should be centred around the patient´s needs and preferences [34]. To adopt a person-centred approach, the patient must be acknowledged, invited and involved [34, 35]. Patients are allowed to express their thoughts and concerns to a healthcare professional who actively listens and validates their experiences [34].

When some patients in our study wanted to talk, they actively chose who they would talk to, whether it was a family member, a friend, or someone on the healthcare staff. While some patients might not have considered nurses as their preferred conversational partners, for others, they were indispensable. This preference could evolve depending on the topic and situation [36]. Therefore, nurses and other health care professionals need to remain receptive and mindful of the ongoing, intricate and context-dependent nature of such interactions [23, 36].

The process of choosing which nurse to talk to involves considering trust and confidence. Patients value trust in their relationships with nurses, regardless of the duration of their acquaintance. Studies by Ikander [37] emphasise the importance of establishing a relationship with nurses, and continuity of care, as highlighted by Engel [23], can facilitate conversations raised when living with the awareness that time is limited due to illness or advanced age.

In our study, patients viewed confidential conversations with nurses as valuable respites. When they engaged with attentive nurses, they felt validated and experienced relief. Participants emphasised the importance of mutual sharing during these interactions. Feeling valued and autonomous can be healing, fostering a return to one’s former self [38]. An authentic meeting reflects the patient’s human worth [39], aligning with a person-centred approach [7]. Patients noted various responses indicating interest and effort from nurses. Seemingly insignificant, small nursing actions can profoundly impact the patient’s well-being and sense of connection [40]. When nurses showed interest, patients felt validated and met with compassion. Establishing a compassionate relationship facilitates communication with patients facing life-threatening illnesses [41, 42]. This caring relationship encompasses respect for patients’ autonomy, dignity and individual needs [38].

The nurse’s openness to the patient’s fragility and suffering may be a result of the ontological understanding of life. The encounter with suffering entails an ethical demand on the nurse, and this is an awareness and a call to relate to the basis of influence we have on each other [43]. By cultivating compassion as the cornerstone of care, nurses can create spaces of safety, trust and meaningful connection [40] for patients nearing the end of life. In the meeting, the nurse can recognise and honour the individual’s wholeness with dignity, to imagine them in a state of well-being and their wishes and desires [8, 9, 40]. The sense of being acknowledged and validated in confidential conversations serves as a vital form of support, offering respite and confirmation.

However, acknowledging the complexities of communication dynamics within these contexts is essential. Not all nurses may feel adequately equipped for or comfortable in such discussions [44]. Our study revealed that some patients experienced nurses who hesitated to engage in confidential conversations. The reluctance of some nurses to broach these sensitive topics may stem from various factors, including personal discomfort, lack of training or experience, or fear of causing emotional distress to the patient [34]. Rattner [45] investigated nurses’ feelings concerning intractable “nonphysical suffering” (emotional, psychological, spiritual and existential). The fact that they could not alleviate patients’ nonphysical suffering was difficult to deal with, and one solution was to ignore them instead. According to Rattner [46], despite the discomfort of being unable to help, healthcare professionals must dare to be present and validate the patient’s experiences. Otherwise, we close the door and suffering goes unspoken. In confidential conversations, nurses can affirm and support patients with courage, presence and time. However, factors such as understaffing and systemic barriers may hinder them from dedicating sufficient time, leading to feelings of inadequacy and moral distress [47]. Nevertheless, healthcare institutions must address these challenges.

In our study, patients who expected attentive listening and support from nurses but found these expectations unmet reported feeling disappointment and loneliness. Mirroring our findings, Tarbi et al. [38] highlighted how the absence of nurse connection can lead to feelings of rejection and isolation, contributing to existential loneliness. Moreover, limited healthcare support may hinder patients in addressing existential challenges, potentially leaving them feeling undervalued [39]. Arman et al. [48] suggested that a care relationship resulting in patient rejection and silence can increase existential suffering.

Neglect and indifference to patient needs, as described by Engel et al. [23], may increase feelings of guilt and shame among patients, rendering them vulnerable, as confirmed by previous studies [39, 48, 49]. Consequently, patients in our study may withdraw from interactions with nurses and remain silent. Regardless of its origin, unsatisfactory care prompts ethical consideration by healthcare personnel, touching on the right to autonomy [50].

Rattner [45] emphasised the importance of understanding that when patients do not talk, it does not mean that they do not want to talk. Since we do not know the reason why the patient does not want to talk, we can, with a person-centred approach, ensure that the opportunity for conversation is created. Tornöe et al. [51] suggested that nurses’ willingness to be present and cultivate silence can embolden them to remain engaged in conversations, encouraging patients to open up and find a moment of respite. By knowing this and through active listening, nurses can identify patient needs to minimise the risk of leaving them in existential loneliness. The patients in our study chose not to talk even if it could increase their suffering.

We discovered various reasons why a patient may choose not to engage in conversation. Apart from feeling unheard, this reluctance could stem from a lack of necessity to talk. Some patients chose not to talk as a means of introspection and self-preservation, finding solace in their thoughts and feelings. This choice may have stemmed from a reluctance to verbalise deeply personal matters or a desire to maintain control over their vulnerability [52]. Respecting patients’ decisions regarding communication was crucial in these instances, as part of a person-centred approach [10]. The realisation of limited time and the uncertainty of when death would occur led patients to attempt to navigate the situation through different strategies. Not talking could be a way of handling the difficulty [2, 38]. Folkman et al. [53] noted that various forms of distraction and avoidance were ways of handling one’s situation. Distraction could be effective in dealing with particularly intense emotions and help reduce distress; however, avoiding thoughts and feelings associated with an event may risk increasing an individual’s anxiety over time [53]. With this understanding, nurses can recognise that patients handle their situations differently, with some preferring not to engage in conversation. By prioritising person-centred care, nurses can approach each patient individually, without imposing expectations, and embrace the diversity in their responses. By remaining emotionally and existentially attuned, nurses can acknowledge and validate the patient’s need for conversation and support.

The patients in our study spoke about their experiences of suffering when they were not treated as a whole person or their support needs were not met. Nurses can thus relieve suffering just by their presence and compassion, and confidential conversation can be one method. Nurses being open and responsive to patients’ ethical demands could be part of good care [40]. By actively listening, nurses can identify patients’ needs, preferences, fears and pains. Placing the patient at the forefront and ensuring they are treated with dignity and respect lays a foundation for person-centred care [34].

### Limitations and methodological considerations

In our study’s methodology, we acknowledge the potential influence of the interview questions on patient responses. Despite efforts to design open-ended questions, certain prompts may inadvertently shape answers, potentially biasing the collected data.

During interviews, we maintained a reflective stance, continuously examining assumptions to minimise misinterpretations. Participants were encouraged to guide discussions and freely express their experiences in a supportive environment, facilitated by active listening techniques [54].

Including dying patients in research was crucial for evidence-based care and understanding their perspectives [32, 33]. Challenges arose due to illness progression, leading to omission in some cases. Despite sample size limitations, the participants had profound experiences, enabling in-depth analysis.

In the analysis, we were vigilant about potential biases. The researchers independently coded transcripts and discussed interpretations to minimise individual interpretations. Seeking alternative perspectives enriched our understanding. Despite our inevitable perspectives, we prioritised methodological rigour. Through reflexivity, open-ended discussions and rigorous analysis, we aimed for valid and reliable findings.

We faced difficulties asking about existential support because participants found it hard to understand and relate to their experiences. After two interviews, we omitted the question from subsequent interviews. Instead, we focused conversations on exploring the diverse range of experiences that participants had. This approach allowed us to capture the richness and complexity of their interactions and perceptions.

## Conclusion

The patients with palliative care needs in our study had different preferences and wishes regarding confidential conversations. Confidential conversations with nurses can offer patients solace, validation and empowerment and serve as an act of validation and existential affirmation, offering comfort amid vulnerability. Patients, discerning in their choice of confidants, prioritise trust and understanding in their relationships. Respecting their autonomy in initiating confidential conversations is essential, acknowledging their individual preferences and the need for personal integrity.

Nurses hold a crucial role in empowering patients to voice their concerns and preferences, especially during confidential conversations. Trustworthiness, familiarity and continuity are essential because patients rely on nurses to be attentive and accessible. Healthcare institutions and universities must invest in comprehensive training and resources to equip nurses with the necessary skills for confidential communication. Offering continuous support, including supervision and opportunities for reflection, enables nurses to evaluate their approach and avoid unintentionally contributing to patients’ feelings of rejection or isolation. Through a person-centred approach, patients’ unique preferences and the importance of maintaining personal integrity can be acknowledged and honoured – regardless of whether, how and when they want to have a confidential conversation.

### Electronic supplementary material

Below is the link to the electronic supplementary material.

Supplementary Material 1

## Data Availability

The datasets used and analysed during the current study are available from the corresponding author under the prerequisite that no sensitive, personal, or confidential data is revealed.

## Abbreviations

no: Number
