# Supplementary material for: “Unless someone sees and hears you, how do you know you exist?” Meanings of confidential conversations – a hermeneutic study of the experiences of patients with palliative care needs
Source: BMC Nurs. 2024 May 18;23:336. doi: 10.1186/s12912-024-01988-9 (PMC11102614; doi:10.1186/s12912-024-01988-9)
Supplement: Supplementary file 1 — Supplementary Material 1 [file 12912_2024_1988_MOESM1_ESM.docx]

*Supplementary file 1*

Interview Guide Study II

Introduction:

We are a research group that has taken an interest in a conversation that we have chosen to call confidential conversation.

The confidential conversation occurs between a patient/person and a nurse and occurs in the moment. The conversation takes place on the patient's initiative and can be about something that needs to be talked about. The conversation often takes place in connection with some other nursing task.

We are now curious about your experience of confidential conversations.

- Please, tell me about an occasion when you felt you had a confidential conversation with a nurse.
  - If no conversation/not with a nurse- can you tell me more about that?
- (Please, tell me about a situation where you perceived a confidential conversation as an existential support?) Interview 1 and 2

Note to self:

The story in focus!

Active reflection

Help questions:

- Can you elaborate?

- What's the meaning of…

- What does it mean?

- How did you experience it?

Demographic questions

Date:

Initials:

Age:

Location:

Man O

Woman O

Other/do not wish to state O

Underlying illness

Cancer O

Cardiovascular disease O

Pulmonary disease O

Neurological disease O

Other O

Current care

Specialized palliative inpatient care O

Palliative Care Unit O

Hospice O

Own home, supported by specialized

palliative home health care O

ASIH/SSIH (advanced home health care) O

In collaboration with municipal home health care O

ECOG: *

0. O

1. O

2. O

3. O

4. O

* The Eastern Cooperative Oncology Group (ECOG) score (published by Oken et al 1982), also called WHO functional level status. Assessment is made by the interviewer together with the nurse in charge before the interview.

Functionality level/functionality status

0 Asymptomatic – able to perform normal activity without restrictions

1 Can't handle physically demanding work. Completely up and running.

2 Able to care for themselves, but not work. Up for more than half the day.

3 Able to partially self-care. Bedridden or wheelchair-bound for more than half the day.

4 Unable to take care of oneself. Completely bed or wheelchair-bound.
